# UBE2C is overexpressed in ESCC tissues and its abrogation attenuates the malignant phenotype of ESCC cell lines

**DOI:** 10.18632/oncotarget.11674

**Published:** 2016-08-29

**Authors:** Antonio Palumbo, Nathalia Meireles Da Costa, Marco De Martino, Romina Sepe, Simona Pellecchia, Vanessa Paiva Leite de Sousa, Pedro Nicolau Neto, Cleber Dario Kruel, Anke Bergman, Luiz Eurico Nasciutti, Alfredo Fusco, Luis Felipe Ribeiro Pinto

**Affiliations:** ^1^ Programa de Carcinogênese Molecular, Instituto Nacional de Câncer - INCA, Rio de Janeiro, Brazil; ^2^ Laboratório de Interações Celulares, Instituto de Ciências Biomédicas, Universidade Federal do Rio de Janeiro Prédio de Ciências da Saúde - Cidade Universitária, Ilha do Fundão, Rio de Janeiro, Brasil; ^3^ Istituto di Endocrinologia e Oncologia Sperimentale - CNR c/o Dipartimento di Medicina Molecolare e Biotecnologie Mediche, Università degli Studi di Napoli “Federico II”, Naples, Italy; ^4^ Departamento de Cirurgia, Faculdade de Medicina da Universidade Federal do Rio Grande do Sul, Porto Alegre, Rio Grande do Sul, Brazil

**Keywords:** esophageal squamous cell carcinoma, UBE2C, diagnostic biomarker, cyclin B1, cell cycle

## Abstract

The esophageal squamous cell carcinoma (ESCC) is widely known as a highly lethal and poor understood cancer, then requiring the search for novel molecular markers to improve its management and patients survival. Recently, ubiquitin-conjugating enzyme E2C (UBE2C) has been figuring as a prominent tumor biomarker candidate, once it has been recognized as a key player in cell cycle progression. In this way, the aim of this study was to evaluate the expression profile of UBE2C gene and protein in ESCC samples, as well as its diagnostic and prognostic marker potential, and its contribution to ESSC genesis and/or progression by performing *in vitro* functional assays. The analysis of *UBE2C* gene expression in 52 paired ESCC samples (tumor and respective histologically normal surrounding tissue), by qRT-PCR, revealed that this gene is overexpressed in 73% of ESCC samples. Subsequently, immunohistochemical analysis confirmed that UBE2C protein expression was upregulated in all ESCC cases, but absent in the histologically normal tumor surrounding tissues. Moreover, we showed that *UBE2C* mRNA expression was able to accurately discriminate ESCC tissue from both healthy esophageal and histologically normal tumor surrounding tissues, pointing out its role as a diagnostic marker for this cancer. Finally, we report that UBE2C affects proliferation rates and cell cycle profile of ESCC cell lines, by directly interfering with cyclin B1 protein levels, suggesting its involvement in crucial steps of ESCC carcinogenesis.

## INTRODUCTION

The esophageal cancer (EC) figures as a highly lethal tumor that responds for the eighth position in cancer incidence worldwide [1]. The esophageal squamous cell carcinoma (ESCC) represents the most common esophageal cancer histotype, being its incidence specially elevated in developing countries [2]. A known correlation between the high lethality displayed by ESCC and its late stage detection has been related as one of the main causes that contribute to the unsuccessful treatment in this disease management [3]. In addition, despite the evolution in cancer detection and treatment [4, 5], the molecular alterations involved in the ESCC carcinogenesis remain poorly understood [3]. Therefore, the identification of new biomarkers and the definition of their roles in the ESCC development could greatly contribute to the improvement of disease diagnosis and therapy.

The ubiquitin conjugating enzyme E2 (UBE2C) is associated with the anaphase promoting complex/ciclossomo (APC/C) and it has been reported that, in addition to its role of antagonizing deubiquitinases such as USP44, UBE2C also promotes the degradation of mitotic cyclins, particularly cyclin B1, during metaphase/anaphase transition [6, 7]. Furthermore, UBE2C is involved in the dissociation of sister chromatids in late mitosis, by inducing the degradation of securin, an enzyme which prevents the chromatids segregation catalyzed by separase enzyme [8]. Therefore, the role of UBE2C in M phase of the cell cycle, particularly in the regulation of the mitotic spindle, does not contribute only to cell cycle progression, but also plays a crucial role in genetic stability [9]. The *UBE2C* gene expression is low in healthy tissues [10], whereas it has been found abundant in several cancer tissues, including ovary [11], prostate [12], breast [13], thyroid [14], lung and uterus [10] carcinomas. Moreover, it has been already shown that high UBE2C expression is also related with a highly malignant phenotype and a poor survival suggesting its role in cancer progression [12, 15, 16, 17, 18].

Due to the fact that ESCC lacks deep molecular knowledge, especially regarding reliable molecular markers of disease diagnosis and evolution, the aim of our study was to evaluate the expression of UBE2C gene and protein in ESCC as possible diagnostic and prognostic marker and its contribution to ESSC carcinogenesis by functional studies *in vitro*. Our results show that UBE2C is highly expressed in ESCC samples, but not in normal mucosa, and that its abrogation is capable of altering proliferation and cell cycle profile of ESCC cell lines, by directly interfering with cyclin B1 protein levels.

## RESULTS

### Clinicopathological features

The clinicopathological characteristics of the 52 ESCC patients evaluated in this study are presented in Table 1. The median age of patients was 59 years, ranging from 39 to 79 years, male patients represented 76.9% of cases and near 65% of all patients were alcohol consumers and/or smokers. The follow up period was of 60 months and the overall survival was of 30.8%. Most of the cases were represented by tumors located at the middle third (57.7%), followed by the upper (26.9%) and the lower third of the esophagus (15.4%). The tumor was detected in 50% of the patients only at the most advanced stages (II or IV), and poorly or moderately differentiated tumors represented 96.2% of cases.

**Table 1:** Clinicopathological characteristics of the 52 esophageal squamous cell cancer (ESCC) patients comprised in the study. N/A = not informed

Clinicopathological features	Frequency
**Age**	
Median (years)	59 (39 - 79)
<59	25 (48%)
≥59	27 (52%)
* Total*	*52*
**Gender**	
Male	40 (76.9%)
Female	12 (23.1%)
* Total*	*52*
**Smoking**	
Ex smoker	9 (17.3%)
No smoker	8 (15.4%)
Smoker	33 (63.5%)
N/A	2 (3.8%)
* Total*	*52*
**Alcohol consumption**	
Ex alcoholic	10 (19.2%)
No alcoholic	8 (15.4%)
Alcoholic	32 (61.5%)
N/A	2 (3.8%)
* Total*	*52*
**Death**	
No	16 (30.8%)
Yes	36 (69.2%)
* Total*	*52*
**Tumor Site**	
Lower Third	8 (15.4%)
Medium Third	30 (57.7%)
Upper Third	14 (26.9%)
* Total*	*52*
**Clinical Stage**	
I	2 (3.8%)
II	15 (28.9%)
III	20 (38.5%)
IV	6 (11.5%)
N/A	9 (17.3%)
* Total*	*52*

The overall survival association with all clinicopathological data was performed and a significant association between anatomical site with overall survival (p=0.018) was observed (Supplementary Table S1).

### ESCC tissues display high levels of UBE2C transcript and protein

**Figure 1:**
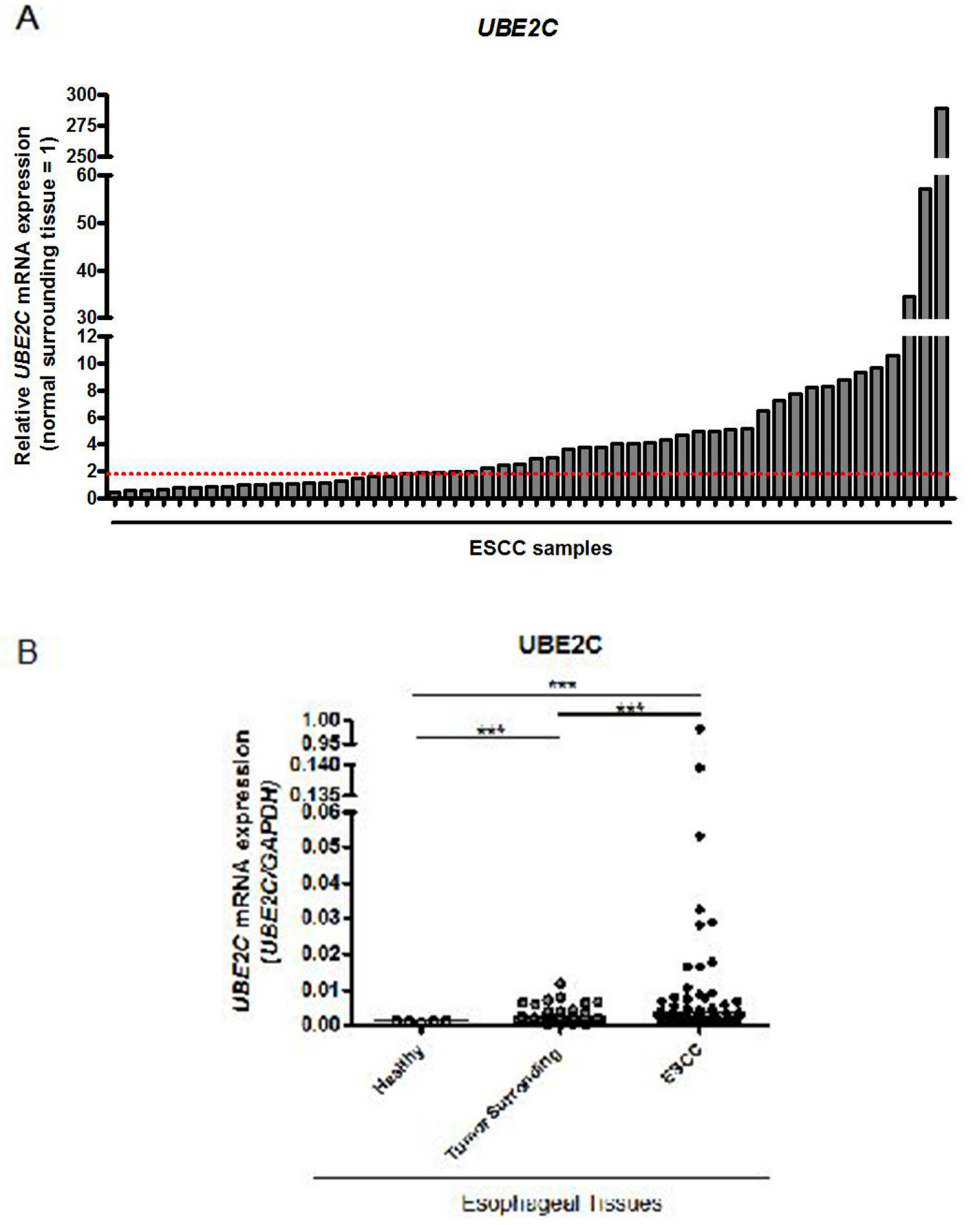
*UBE2C* mRNA expression pattern in esophageal squamous cell carcinomas (ESCC) **A.** qRT-PCR analysis of *UBE2C* mRNA levels in 52 paired ESCC samples. Values are expressed as relative to those obtained in tumors respective histologically normal surrounding tissue (=1). ESCC samples presenting over 2-fold increase (red dashed line) in relative *UBE2C* expression were considered upregulated. **B.** qRT-PCR evaluation of *UBE2C* mRNA levels distribution in the groups of healthy (n=5), histologically normal surrounding (n=52) and their paired ESCC (n=52) tissues. *UBE2C* mRNA levels were normalized by those of *GAPDH*, used as the housekeeping gene.

In order to analyze the profile of *UBE2C* mRNA expression in tumor and non-tumor esophageal tissue, we evaluated its expression in 52 ESCC paired samples (tumor and histologically normal surrounding tissue) and 5 samples of normal esophageal tissue from healthy subjects by qRT-PCR. We observed that 73% of ESCC samples analyzed presented an increase in *UBE2C* gene expression when compared to their respective normal surrounding tissue counterparts (Figure 1A). The relative *UBE2C* expression values of ESCC samples, when compared to their paired normal surrounding tissue, ranged from 0.3 to 289-fold change, being the median value of 2.5-fold change. Additionally, we evaluated the distribution of *UBE2C* mRNA expression levels in the groups of healthy esophageal tissues, ESCC samples and tumor surrounding mucosa, being the mRNA levels median values of 0.0011; 0.0019 and 0.0039, respectively. Further, the levels of *UBE2C* expression detected in ESCC group were significantly higher than those found in the other groups, being its median expression value approximately 3.5- and 2.0-fold higher than those found in healthy and tumor surrounding esophageal tissue groups, respectively (Figure 1B). Furthermore, the median value of *UBE2C* mRNA expression levels observed in the tumor surrounding mucosa group was also significantly higher than that of the healthy esophageal tissues group (Figure 1B).

Statistical analysis of the association of *UBE2C* gene expression and all the clinicopathological data was performed and no significant association was observed (Supplementary Table S2). Moreover, no statistically significant correlation between *UBE2C* overexpression and ESCC patients overall survival was detected (Supplementary Table S3).

**Figure 2:**
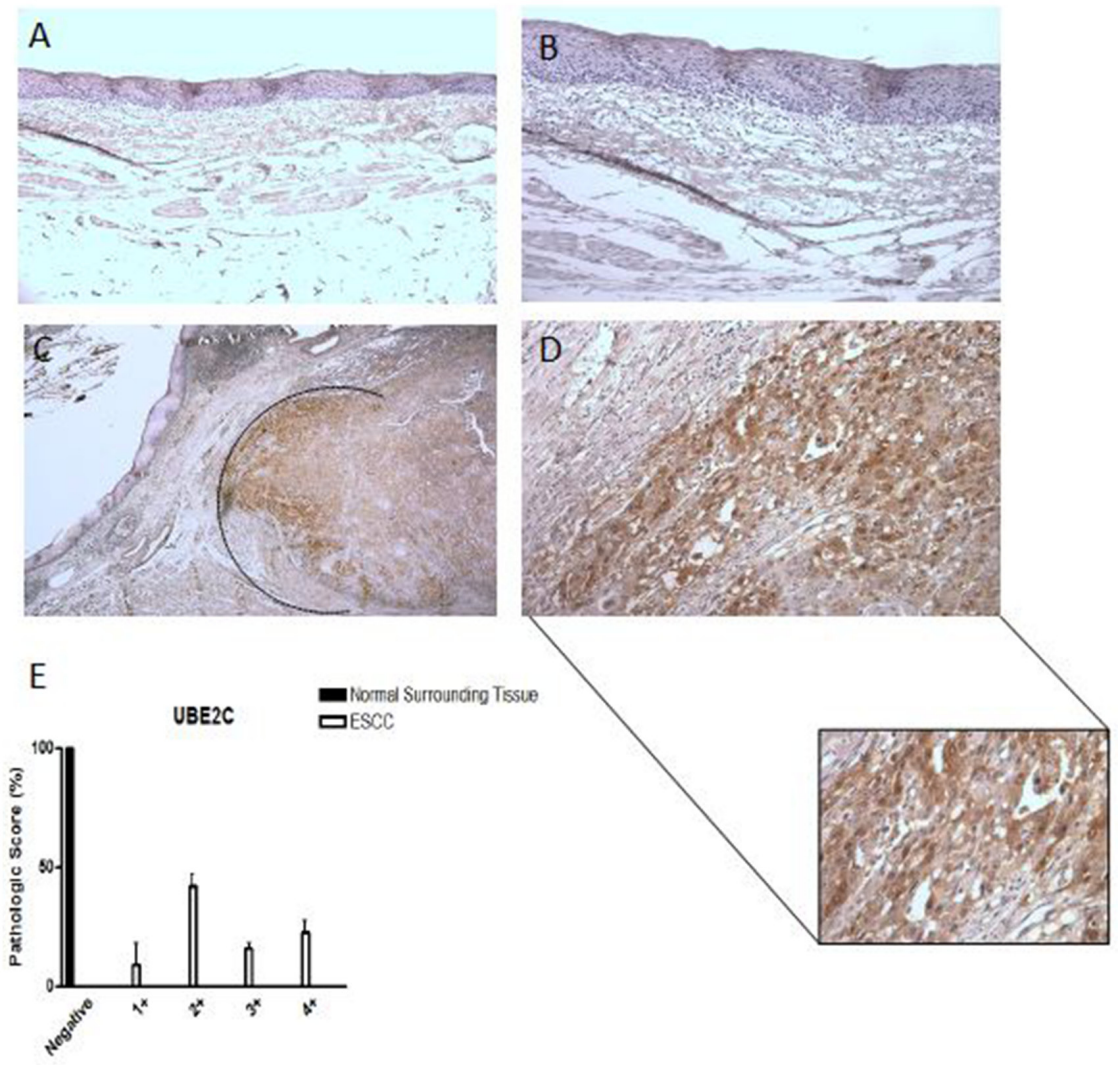
UBE2C protein expression pattern in esophageal squamous cell carcinomas (ESCC) Representative immunohistochemistry micrographs of histologically normal surrounding mucosa showing negative staining for UBE2C **A.** – 100X magnification and **B.** – 200X magnification) and of ESCC sample positively stained for UBE2C **C.** – 25X magnification and **D.** – 200X magnification). In the detail, a zoom of the ESCC invasion front, highly positive for UBE2C staining. **E.** Graphical representation of the 22 ESCC samples and their respective normal surrounding mucosas staining score.

Next, we evaluated UBE2C protein expression in 22 paired ESCC samples by immunohistochemistry. We observed a nuclear and cytoplasmic immunostaining in all ESCC cases. UBE2C expression was particularly present in tumor foci, especially in the tumor invasive front (Figure 2C and 2D) where the intensity of UBE2C immunostaining was very high. On other hand, UBE2C protein was not detected in tumor surrounding tissue samples (Figure 2A and 2B). Finally, 50% of the analyzed ESCC samples were scored as grade 1+ and 2+ and the remaining 50% as score grade 3+ and 4+ (Figure 2E), regarding UBE2C expression levels pathological score. These data, according to those obtained on *UBE2C* mRNA expression by qRT-PCR, confirm that UBE2C is overexpressed in the ESCC tissue, when compared to both healthy esophageal tissues and tumor surrounding counterparts.

### *UBE2C* mRNA expression distinguishes between ESCC and non-tumor esophageal tissues with high sensitivity and specificity

**Figure 3:**
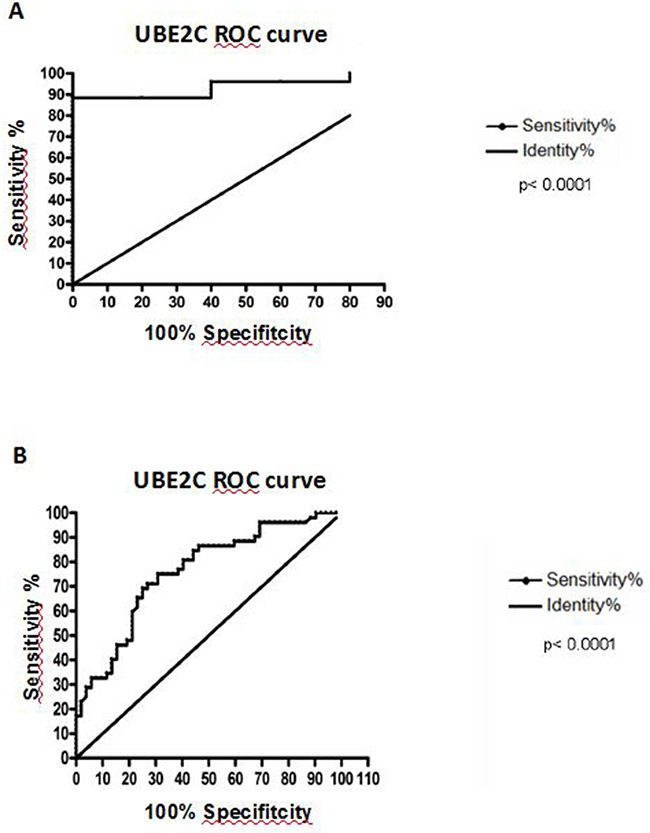
Receiver Operating Characteristc (ROC) analysis **A.** Discrimination of healthy esophageal tissue (n=5) from ESCC samples with 88.46% of sensitivity and 100% of specificity, at a cut-off point of 0.001354 (area under the curve = 0.9385). **B.** Discrimination of histologically normal surrounding mucosa (n=52) from their paired ESCC samples, being the area under curve (AUC) = 0.7605; Sensitivity = 71.15% and Specificity = 73.08%, at a cut-off point of 0.0002343 (B). Both curves are relative to *UBE2C* mRNA expression.

Aiming to evaluate whether *UBE2C* mRNA expression would be able to discriminate between tumor and non-tumor esophageal tissues, we performed the Receiver Operating Characteristc (ROC) curve using the gene expression values from the healthy esophageal tissues, tumor surrounding tissues and ESCC groups. The expression of *UBE2C* was able to accurately discriminate ESCC samples from normal (p<0.0001) and tumor surrounding tissues (p<0.0001), with sensitivity and specificity of, respectively, 88.46% and 100% (discrimination between ESCC and healthy esophageal tissue) and 71.15% and 73.08% (discrimination between ESCC and normal surrounding tissue) (Figure 3).

These results suggest that *UBE2C* mRNA expression levels can be envisaged as a diagnostic marker for ESCC.

### The inhibition of UBE2C expression alters growth and cell cycle profile of two ESCC cell lines

**Figure 4:**
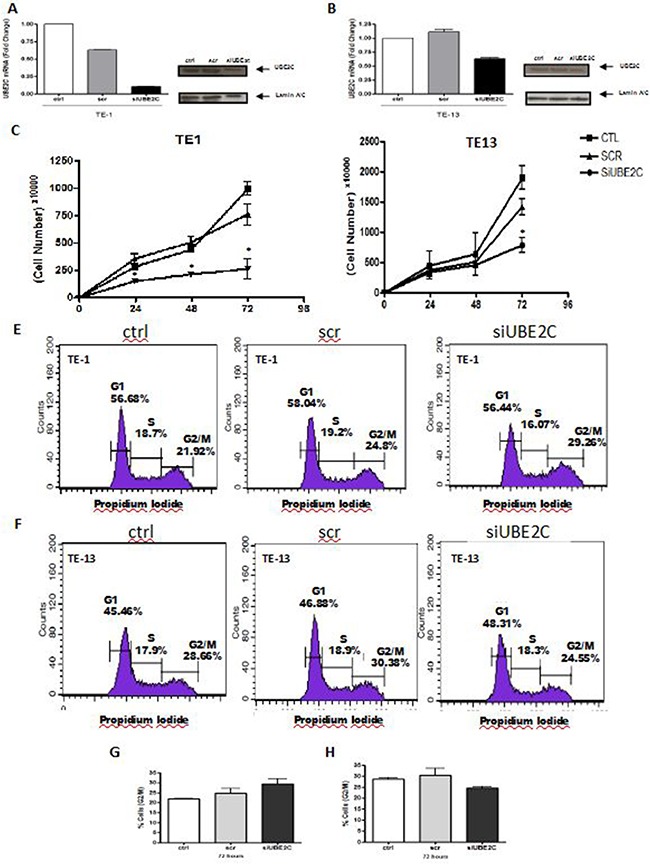
UBE2C silencing alters growth rates and cell cycle profile of ESCC cell lines Analysis of UBE2C mRNA and protein levels by qRT-PCR and western blot, respectively, in TE-1 **A.** and TE-13 **B.** ESCC cells transfected with siRNA targeting UBE2C (siUBE2C) compared to those non-transfected (ctrl) and transfected with a siRNA scrambled sequence (scr). Proliferation rates and cell cycle profile ofTE-1 **C.** and **E.** TE-13 (**D** and **F.** cells transfected with siRNA targeting UBE2C (siUBE2C) compared to those non-transfected (ctrl) and transfected with a siRNA scrambled sequence (scr). Graphical representation of the percentage of TE-1 **G.** and TE-13 **H.** cellsin G2/M phase of the cell cycle analysis represented in **Figures 4E and 4F**. * p < 0.005

Since we observed that UBE2C transcript and protein are overexpressed in ESCC samples, we decided to investigate the role of UBE2C in esophageal carcinogenesis by performing UBE2C transient knockdown in ESCC cell lines TE-1 and TE-13. To this end, a small interfering RNA (siRNA) targeting UBE2C transcript was transiently transfected into TE-1 and TE-13 cell lines. Following UBE2C siRNA transfection, we firstly evaluated the UBE2C siRNA transfection efficiency by flow cytometry, being observed an efficiency rate of 88.58% and 84.35% in TE-1 and TE-13 cells, respectively (Supplementary Figure S1). Next, 72 hours after UBE2C knockdown, its depletion was confirmed by qRT-PCR and Western blotting. Indeed, a great inhibition of UBE2C transcript and protein expression in TE-13 and, particularly, in TE-1 cells was observed (Figure 4A and 4B).

Since UBE2C is involved with G2/M transition during cell cycle progression, TE-1 and TE-13 cells were synchronized at G1 phase of cell cycle, by the treatment with Thymidine, prior the evaluation of the *in vitro* consequences of UBE2C inhibition (Supplementary Figure S2). Following synchronization and UBE2C silencing, the growth rates of TE-1 and TE-13 cells were evaluated along 72 hours and, as shown in Figure 4C and 4D, TE-1 cells transfected with UBE2C siRNA showed a statistically significant decrease in their proliferation rates at all the time intervals analyzed, when compared with both control cells or cells transfected with scrambled siRNA (Figure 4C). The effect of synchronization and UBE2C abrogation on growth rates was less pronounced in TE-13 cells which presented a statistically significant decrease in their proliferation only 72 hours after UBE2C knockdown, when compared with both control cells and cells transfected with scrambled siRNA (Figure 4D). Next, we investigated the cell cycle profile of the ESCC cell lines, TE-1 and TE-13, following their synchronization and UBE2C depletion. TE-1 cells lacking UBE2C were slightly arrested at G2/M phases of the cell cycle, when compared with both control cells and scrambled siRNA-transfected cells (Figure 4E and 4G), nevertheless, this phenomenon was not observed for TE-13 cell line (Figure 4F and 4H). Together, these results show that UBE2C silencing is capable of altering crucial cellular features, such as proliferation and cell cycle profile, and these effects are more evident in TE-1 cells.

### Cyclin B1 expression is enhanced by UBE2C knock down in ESCC cell lines

**Figure 5:**
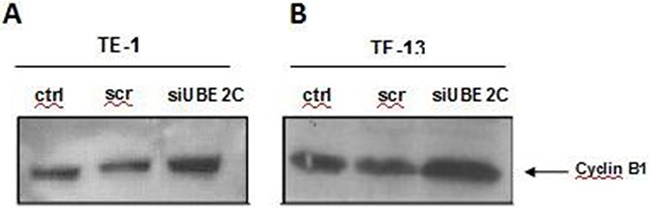
UBE2C silencing leads to upregulation of Cyclin B1 in ESCC cell lines Western blot analysis of Cyclin B1 protein expression in ESCC cell lines TE-1 **A.** and TE-13 **B.** transfected with siRNA targeting UBE2C (siUBE2C) compared to those non-transfected (ctrl) and transfected with a siRNA scrambled sequence (scr). Lamin A/C expression was used as a loading control and is demonstrated in Figure 4A and 4B.

Cyclin B1 is a key protein involved with progression of cell cycle and its degradation promoted by APC/C complex, particularly by UBE2C, represents a crucial step in the G2/M transition. Therefore, in order to understand the decrease observed in cell growth rates and the changes detected in the cell cycle profile of the UBE2C-knocked down TE-1 and TE-13 cell line, we analyzed the expression cyclin B1. As shown in Figure 5A and 5B, cyclin B1 expression was strongly increased after UBE2C inhibition in TE-13 and, mainly, in the TE-1 cells, when compared with both untransfected cells and scrambled siRNA-transfected cells.

This result indicates that UBE2C regulates cyclin B1 expression also in ESCC cell lines, and this may be one of the mechanisms by which it controls their cell cycle and proliferation.

## DISCUSSION

The ESCC is widely known as a highly lethal and poorly understood cancer, being its late detection associated with the high death rates observed in its patients. Therefore, the search for novel molecular markers could promote the development of new strategies in the ESCC management. In this sense, it has been reported that not only the expression, but also the crucial role played by ubiquitin conjugating enzyme UBE2C in the cell cycle progression are altered in several types of cancer [10–14]. Therefore, the aim of the present work has been to analyze the role played by UBE2C in the development of ESCC. Then, we analyzed the impact of UBE2C expression on the diagnosis, prognosis and progression of ESCC by using translational and *in vitro* experimental approaches.

First, an increased expression of *UBE2C* transcript was observed in approximately 70% of the ESCC samples studied when compared with their respective histologically normal surrounding tissue. Moreover, *UBE2C* median gene expression level detected in ESCC samples was approximately 10-fold higher than those of the normal and tumor surrounding esophageal tissue samples. Furthermore, immunohistochemical analysis of UBE2C protein corroborated the gene expression data, once UBE2C protein was quite abundant in most of the ESCC samples, whereas it was completely absent in tumor surroundingtissues. Our results are in accordance with the data already present in the literature, since UBE2C overexpression has been reported in several distinct tumor types [13, 19, 20, 21]. Moreover, UBE2C overexpression has been associated with decreased survival rates of the patients in a wide range of tumors, including the esophageal adenocarcinoma [12, 15, 16, 17, 18, 22].

Contrary to the above cited studies, we did not detect statistically significant impact of *UBE2C* expression on the survival of the ESCC patients included in our study, neither association between *UBE2C* expression and any of the evaluated clinicopathological features. These results can be due to the fact that most of the samples included in the present study are represented by poor or moderately differentiated ESCC and mainly come from advanced stages. Therefore, our sample profile lacks representation of the earlier molecular events occurring in ESCC development, and then does not allow to detect association with clinicopathological features.

Even though our results do not support the high *UBE2C* as a prognostic marker, a diagnostic potential for UBE2C detection for ESCC must be taken in consideration, since *UBE2C* mRNA expression values were capable of clearly discriminating ESCC tissues from both tumor surrounding and healthy esophageal tissues. According to the results reported here, UBE2C expression pattern has been already proposed as a promising molecular tool for the diagnosis of several tumor types [8, 23–27].

Finally, to understand the role of UBE2C in ESCC progression, we conducted a series of *in vitro* functional experiments. First, after the transient knockdown of UBE2C in the ESCC cell lines TE-1 and TE-13, we observed a significant decrease in TE-1 cell growth, at all time intervals analyzed during 72 hours, while in TE-13 cells, the statistically significant difference was observed only at the time interval of 72 hours.

Since UBE2C is involved in cell cycle progression, particularly in the metaphasis to anaphasis transition regulating the G2/M transition by enhancing degradation of cyclin B1 [28–30], we performed FACS analysis of UBEC2 siRNA-transfected TE-1 and TE-13 cells, and analyzed the cyclin B1 expression in the same cells. Accordingly, cell cycle analysis revealed a higher percentage of UBE2C-silenced TE-1 cells in G2/M phases with respect to the control cells. However, the same result was not observed in TE-13 cell line. Moreover, the cell lines TE-1 and TE-13 knocked down for UBE2C showed an increased expression of cyclin B1, that was more pronounced in TE-1 cells, in comparison to the cell lines transfected with a scrambled oligonucleotide and untransfected cells.

As known, the Cyclin B1 destruction represents a molecular change in the cell cycle control, once the progression of G2 phase to mitosis is promoted by its degradation, instead the regular increase in the cyclins expression that normally control the cell cycle progression [28–30]. Therefore, our results suggest that the reduced growth rates observed in the ESCC cell lines upon UBE2C silencing might be a consequence of the arrest in G2/M phase of cell cycle, that is induced by a reduced degradation of cyclin B1, caused by the suppression of UBE2C expression, that was particularly observed in TE-1 cell line. On the other hand, the different results obtained by UBE2C silencing in the TE-13 cell line may be due to a less efficient inhibition of UBE2C transcript and protein in these cells, when compared with the TE-1 cells.

Finally, the knockdown of UBE2C in the ESCC cell lines did not result in statistically significant differences in the apoptotic cell death rate (Supplementary Figure S3). This result is quite interesting, once the cell cycle arrest observed in the TE-1 cells didnot result in the apoptosis of these cells, as previously reported by Wagner and colleagues [21]. On the other hand, Jiang and colleagues showed that UBE2C silencing was able to induce apoptosis of U251 glioma cell line, through *TP53* and Bax activation [31]. The absence of cell death after the knockdown of UBE2C in TE-1 and TE-13 cells could be related with the absence of the apoptotic pathway mediated by *TP53* whose loss represents the most frequent molecular alteration in esophageal carcinogenesis [32], and is also present in the ESCC cell lines used in this study [33, 34].

In conclusion, the results reported here support UBE2C as a promising ESCC biomarker, and its key role in the ESCC progression by altering cell cycle progression.

## MATERIALS AND METHODS

### Patients and samples

The gene expression analysis comprised paired biopsies (tumor and histologically normal surrounding tissues, collected at least 5 cm far from the tumor border) obtained from 52 ESCC patients who were submitted to endoscopy, and had confirmed ESCC diagnosis, from 2006 to 2013 at the Brazilian National Cancer Institute (INCA). Epidemiological and clinicopathological data were obtained, respectively, through interviews by using a standardized questionnaire and from patient's medical records. Additional 5 samples of normal esophageal tissue from healthy individuals who underwent endoscopy due to any reason other than cancer at the Hospital Pedro Ernesto (HUPE / UERJ) were also included in this study. For immunohistochemical analysis, another 22 ESCC samples, and their respective normal surrounding mucosa, were collected from patients who underwent surgery at the Hospital das Clínicas de Porto Alegre (HCPA / UFRGS) and at INCA, from 2006 and 2013, fixed in formalin and embedded in paraffin for further analysis. Tumor tissue and their respective adjacent mucosa were removed respecting 5 cm limit from tumor border. None of the patients comprised in this study had undergone any type of chemotherapy and/or radiotherapy. The use of the human samples was approved by the Ethics Committee of the respective institutions (INCA - 115/10, HUPE / UERJ - 416, HCPA / UFRGS - 02 223). All patients and healthy individuals, who kindly agreed to participate in the study, signed a consent form.

### Cell lines and transfections

TE-1 and TE-13 cell lines were derived from ESCC and were kindly provided by Dr. Pierre Hainaut (IARC, France). Both lineages were cultured in RPMI medium (Invitrogen) supplemented with 10% fetal bovine serum (Gibco) and 1% of the cocktail penicillin / glutamine / streptomycin (Invitrogen) and maintained at 37°C under 5% CO_2_. For silencing experiments, 1.5×10^5^ cells were seeded in a 6-well plate. 24 hours prior siRNA transfection, TE-1 and TE-13 cells were treated with Thymidine (Sigma) at the concentration of 4 mM in RPMI medium supplemented with 0.5% FBS (Gibco), in order to synchronize them in G1/S phase of the cell cycle. Then, double-strand siRNA oligonucleotides targeting *UBE2C* gene or fluorescent scrambled control siRNA (#1022563) were transfected into TE-1 and TE-13 cells at a final concentration of 120 nM. All siRNA duplexes were purchased from Qiagen (Qiagen, Valencia, CA) and were transfected by using Lipofectamine 2000 (Invitrogen, Carlsbad, CA), according to the manufacturer's recommendations.

### RNA extraction, reverse transcription and qRT-PCR

Total RNA was extracted from tissue samples and cell lineages using Trizolfi reagent, according to the manufacturer's instructions (Invitrogen). All RNA samples were measured by spectrophotometry and 1 μg of RNA was reverse transcribed by using SuperScript™ II Reverse Transcriptase (Invitrogen), following the manufacturer's protocol. *UBE2C* expression analysis was performed in an Illumina Eco Real-Time PCR System using SYBR Green Master Mix (QiaGen) and oligonucleotides, as follows: *UBE2C* Forward: 5′ TGGTCTGCCCTGTATGATGT 3′, *UBE2C* Reverse: 5′ AAAAGCTGTGGGGTTTTTCC 3′; GAPDH Forward: 5′ CAACAGCCTCAAGATCATCAGCAA 3′, GAPDH Reverse: 5′ AGTGATGGCATGGACTGTGGTCAT 3′. Each reaction consisted of 5.0 μL of Quantifast SYBR Green PCR Master Mix (Qiagen), 10 pmols of primers and 1 μL of cDNA. The amplification reaction was performed as follows: 5 minutes for DNA pre-denaturation at 95°C, followed by 40 cycles of hybridization and complementary chain synthesis for 5 seconds at 95°C and 10 seconds at 60°C. Each sample was analyzed in triplicate. Relative mRNA levels were calculated using the comparative threshold cycle (CT) with the analyzed gene expression levels normalized by those of *GAPDH* and using the normal surrounding tissue as the reference (2^−ΔΔCT^ formula).

### Immunohistochemistry

Immunohistochemistry (IHC) was performed on 3 μm paraffin sections of 22 ESCC cases and their respective normal surrounding mucosa. For UBE2C antigen retrieval, sections were incubated in water bath while submerged in a target buffer solution (DAKO), pH 9.0, for 40 minutes at 98°C. Sections were then incubated with the primary monoclonal antibody against UBE2C (Boston Biochem-A650, working dilution 1:100), during 12 hours, at least. FFPE anaplastic thyroid carcinoma samples served as positive controls of UBE2C staining. As negative control, the primary antibody was replaced by the diluent solution. The detection system used was the NovoLink™ Max Polymer Detection System (Leica Biosystems), following the protocol described by the manufacturer, using diaminobenzidine as substrate - DAKO. Sections were counterstained with Harris' hematoxylin. The staining score evaluation was performed by three independent pathologists. For both proteins, scored cases were considered 1+ when positive staining was present in up to 25% of tumor region; 2+ when staining was present between 26% and 50%, 3+ when staining was present between 51% and 75% and 4+ when staining was present between 76% and 100% of tumor region.

### Protein extraction and western blot

Proteins were extracted from the cells by washing them twice in ice-cold PBS and subsequently lysing them by using RIPA-like buffer (250 mMNaCl, 50 mM TRIS-HCl pH 7.4, 0.1% SDS, 2 mM DTT and 0.5% NP-40) containing protease inhibitors (Complete-Mini, Roche). Protein concentration was determined by the Bradford assay (Bio-Rad) using bovine serum albumin as standard and equal amounts of proteins were resolved onto a 12.5% SDS-PAGE, transferred a to nitrocellulose-membrane (Whatman^®^Protran^®^) and probed with primary antibodiy anti-UBE2C. Membranes were then incubated with the horseradish peroxidase-conjugated secondary antibody (1:10,000) and detection was performed with enhanced chemiluminescence (ECL Kit, Amersham).

### Proliferation assay

Following transfection of TE-1 and TE-13 cells with either siRNA targeting UBE2C or fluorescent scrambled control siRNA, as previously described, cells were counted daily using an automated cell counter (Countess, Invitrogen) during 3 consecutive days to extrapolate growth curves. The values represent means +/− SEM of the three different experiments.

### Cell cycle assay

Cell cycle analysis was performed by using propidium iodide assay. Briefly: following transfection of TE-1 and TE-13 cells with either siRNA targeting *UBE2C* or fluorescent scrambled control siRNA, as previously described, cell pellets were resuspended in 500 μL of propidium iodide solution (PBS, 0.1% Triton X-100, 0.1% RNAse and 50 μg/mL propidium iodide – Sigma) and incubated for 5 min on ice. Cell cycle analysis was assessed by flow cytometry (FACScalibur, BD Bioscience) after the acquisition of 20,000 events and the data were analyzed in Cell Quest software.

### Statistical analysis

Frequencies of clinicopathological data and mRNA expression levels of *UBE2C* were calculated. For continuous variables, we performed a descriptive analysis of central and dispersion tendencies. To assess the relationship between mRNA expression levels and clinicopathological features, we used the Fisher's exact test. The Kaplan-Meier method was used to evaluate overall survival and disease-free survival, based on a statistically significant confidence interval of 95% and p-value < 0.05. Finally, in order to assess the impact of *UBE2C* gene expression profile on overall survival and its statistical significance, Kaplan-Meier Test was performed. For univariate analysis of *UBE2C* mRNA expression impact on ESCC patients survival, the cutoff value defined was the median of its mRNA levels. Patients expressing *UBE2C* mRNA levels above the median were considered as “high tumor expression” and those expressing mRNA levels below the median were considered as “low tumor expression”. Cox regression was performed with all clinicopathological parameters to adjust the effect of clinical stage and age. All statistical analysis was performed with GraphPad Prism 5.0 (GraphPad Software Incorporated, USA) and SPSS 17.0. The final values were considered of statistical significance when p < 0.05. To *in vitro* experiments the statistical analysis was performed using the Graph Pad Prism 5.0 following by Anova e Student t test.

## SUPPLEMENTARY MATERIALS FIGURES AND TABLES



## References

[1] Ferlay J, Soerjomataram I, Ervik M, Dikshit R, Eser S, Mathers C, Rebelo M, Parkin DM, Forman D, Bray F (2013). GLOBOCAN 2012 v1 0 Cancer Incidence and Mortality Worldwide: IARC Cancer Base No 11 [Internet]. http://globocaniarcfr/.

[2] Vizcaino AP, Moreno V, Lambert R, Parkin DM (2002). Time trends incidence of both major histologic types of esophageal carcinomas in selected countries 1973-1995. Int J Cancer.

[3] Costa NM, Soares Lima SC, de Almeida Simão T, Ribeiro Pinto LF (2013). The potential of molecular markers to improve interventions through the natural history of oesophageal squamous cell carcinoma. Biosci Rep.

[4] Palumbo A, Da Costa NM, Bonamino MH, Pinto LFR, Nasciutti LE (2015). Genetic instability in the tumor microenvironment: a new look at an old neighbor. Molecular cancer.

[5] Roshandel G, Nourouzi A, Pourshams A, Semnani S, Merat S, Khoshnia M (2013). Endoscopic screening for esophageal squamous cell carcinoma. Arch Of Iran Med.

[6] Townsley FM, Aristarkhov A, Beck S, Hershko A, Ruderman JV Dominant-negative cyclin-selective ubiquitin carrier protein E2-C/UbcH10 blocks cells in metaphase.

[7] Stegmeier F, Rape M, Draviam VM, Nalepa G, Sowa ME, Ang XL, Kirschner MW (2007). Anaphase initiation is regulated by antagonistic ubiquitination and deubiquitination activities. Nature.

[8] Xie C, Powell C, Yao M, Wu J, Dong Q (2014). Ubiquitin-conjugating enzyme E2C: A potential cancer biomarker. The international journal of biochemistry & cell biology.

[9] van Ree JH, Jeganathan KB, Malureanu L, van Deursen JM (2010). Overexpression of the E2 ubiquitin–conjugating enzyme UbcH10 causes chromosome missegregation and tumor formation. The Journal of cell biology.

[10] Okamoto Y, Ozaki T, Miyazaki K, Aoyama M, Miyazaki M, Nakagawara A (2003). UbcH10 is the cancer-related E2 ubiquitin-conjugating enzyme. Cancer Research.

[11] Berlingieri MT, Pallante P, Guida M, Nappi C, Masciullo V, Scambia G, Ferraro A, Leone V, Sboner A, Barbareschi M, Ferro A, Troncone G, Fusco A (2007). Ubc expression may be a useful tool in the prognosis of ovarian carcinomas. Oncogene.

[12] Wang H, Zhang C, Rorick A, Wu D, Chiu M, Thomas-Ahner J, Wang Q (2011). CCI-779 Inhibits Cell-Cycle G2–M Progression and Invasion of Castration-Resistant Prostate Cancer via Attenuation of UBE2C Transcription and mRNA Stability. Cancer research.

[13] Berlingieri M T, Pallante P, Sboner A, Barbareschi M, Bianco M, Ferraro A, Fusco A (2007). UbcH10 is overexpressed in malignant breast carcinomas. European Journal of Cancer.

[14] Pallante P, Berlingieri MT, Troncone G, Kruhoffer M, Orntoft TF, Viglietto G, Palombini L (2005). UbcH10 overexpression may represent a marker of anaplastic thyroid carcinomas. British journal of cancer.

[15] Cunha IW, Carvalho KC, Martins WK, Marques SM, Muto NH, Falzoni R, Neves EJ (2010). Identification of genes associated with local aggressiveness and metastatic behavior in soft tissue tumors. Translational oncology.

[16] Shen Z, Jiang X, Zeng C, Zheng S, Luo B, Zeng Y, Jie W (2013). High expression of ubiquitin-conjugating enzyme 2C (UBE2C) correlates with nasopharyngeal carcinoma progression. BMC cancer.

[17] Lu J, Wen M, Huang Y, He X, Wang Y, Wu Q, Rodriguez C (2013). A C2ORF40 suppresses breast cancer cell proliferation and invasion through modulating expression of M phase cell cycle genes. Epigenetics.

[18] Fujita T, Ikeda H, Taira N, Hatoh S, Naito M, Doihara H (2009). Overexpression of UbcH10 alternates the cell cycle profile and accelerate the tumor proliferation in colon cancer. BMC cancer.

[19] Pallante P, Malapelle U, Berlingieri MT, Bellevicine C, Sepe R, Federico A, Troncone G (2013). UbcH10 overexpression in human lung carcinomas and its correlation with EGFR and p53 mutational status. European Journal of Cancer.

[20] Jiang L, Huang CG, Lu YC, Luo C, Hu GH, Liu HM, Han HX (2008). Expression of ubiquitin-conjugating enzyme E2C/UbcH10 in astrocytic tumors. Brain research.

[21] Wagner W, Frierson HF, Butz N, Mestan J, Hofmann F, Deveraux QL, Hampton GM (2004). Overexpression genomic amplification and therapeutic potential of inhibiting the UbcH10 ubiquitin conjugase in human carcinomas of diverse anatomic origin. Oncogene.

[22] Lin J, Raoof DA, Wang Z, Lin MY, Thomas DG, Greenson JK, Lin L (2006). Expression and effect of inhibition of the ubiquitin-conjugating enzyme E2C on esophageal adenocarcinoma. Neoplasia.

[23] Kadara H, Lacroix L, Behrens C, Solis L, Gu X, Lee JJ, Lotan R (2009). Identification of gene signatures and molecular markers for human lung cancer prognosis using an in vitro lung carcinogenesis system. Cancer Prevention Research.

[24] Rajkumar T, Sabitha K, Vijayalakshmi N, Shirley S, Bose MV, Gopal G, Selvaluxmy G (2011). Identification and validation of genes involved in cervical tumourigenesis. BMC cancer.

[25] Chen CC, Chang TW, Chen FM, Hou MF, Hung SY, Chong IW, Lin SR (2006). Combination of multiple mRNA markers (PTTG1 Survivin UbcH10 and TK1) in the diagnosis of Taiwanese patients with breast cancer by membrane array. Oncology.

[26] Du H, Jie L, Xu W, Wu Y, Liu T, Li M (2012). A monoclonal antibody against a potential cancer biomarker human ubiquitin-conjugating enzyme E2. Hybridoma.

[27] Guerriero E, Ferraro A, Desiderio D, Pallante P, Berlingieri MT, Iaccarino A, Troncone G (2010). UbcH10 expression on thyroid fine–needle aspirates. Cancer cytopathology.

[28] Chang L, Zhang Z, Yang J, McLaughlin SH, Barford D (2015). Atomic structure of the APC/C and its mechanism of protein ubiquitination. Nature.

[29] Chang L, Barford D (2014). Insights into the anaphase-promoting complex: a molecular machine that regulates mitosis. Current opinion in structural biology.

[30] Sivakumar S, Gorbsky GJ (2015). Spatiotemporal regulation of the anaphase-promoting complex in mitosis. Nature Reviews Molecular Cell Biology.

[31] Jiang L, Bao Y, Luo C, Hu G, Huang C, Ding X, Lu Y (2010). Knockdown of ubiquitin-conjugating enzyme E2C/UbcH10 expression by RNA interference inhibits glioma cell proliferation and enhances cell apoptosis in vitro. Journal of cancer research and clinical oncology.

[32] Mandard A, Hainaut P M, Hollstein M (2000). Genetic steps in the development of squamous cell carcinoma of the esophagus. Mutation Research/Reviews in Mutation Research.

[33] Nishihira T, Hashimoto Y, Katayama M, Mori S, Kuroki T (1993). Molecular and cellular features of esophageal cancer cells. Journal of cancer research and clinical oncology.

[34] Barnas C, Martel–Planche G, Furukawa Y, Hollstein M, Montesano R, Hainaut P (1997). Inactivation of the p53 protein in cell lines derived from human esophageal cancers. International journal of cancer.

