# Supplementary material for: UBE2C is overexpressed in ESCC tissues and its abrogation attenuates the malignant phenotype of ESCC cell lines
Source: Oncotarget. 2016 Aug 29;7(40):65876–87. doi: 10.18632/oncotarget.11674 (PMC5323199; doi:10.18632/oncotarget.11674)
Supplement: Supplementary file 1 [file oncotarget-07-65876-s001.pdf]

UBE2C is overexpressed in ESCC tissues and its abrogation attenuates the malignant phenotype of ESCC cell lines

SUPPLEMENTARY FIGURES AND TABLES

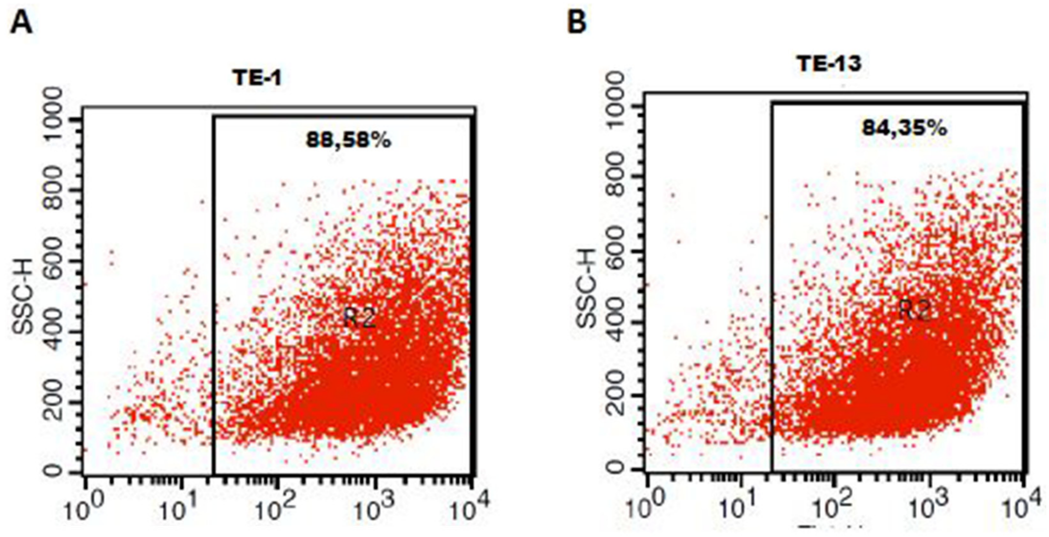

Supplementary Figure S1: siRNA sequences were efficiently transfected into ESCC cell lines. Flow cytometry histograms representing the percentage of TE-1 **A.** and TE-13 **B.** cells transfected with the fluorescent siRNA scrambled sequence (scr).

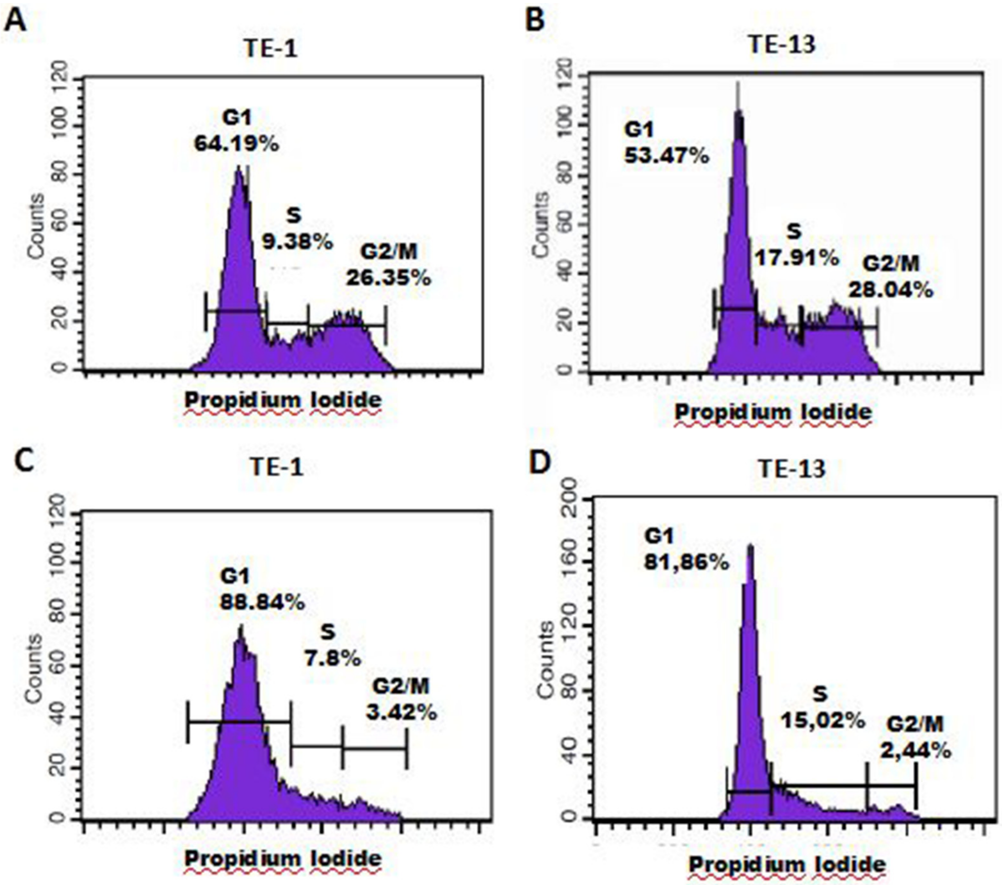

Supplementary Figure S2: ESCC cell lines were synchronized at G1/S phase of the cell cycle following treatment with thymidin. Representative histograms of the cell cycle profile of control TE-1 **A.** and TE-13 **B.** cells and after the 24 hours treatment with 4 mM of thymidine **C.** and **D.**, evaluated by flow cytometry.

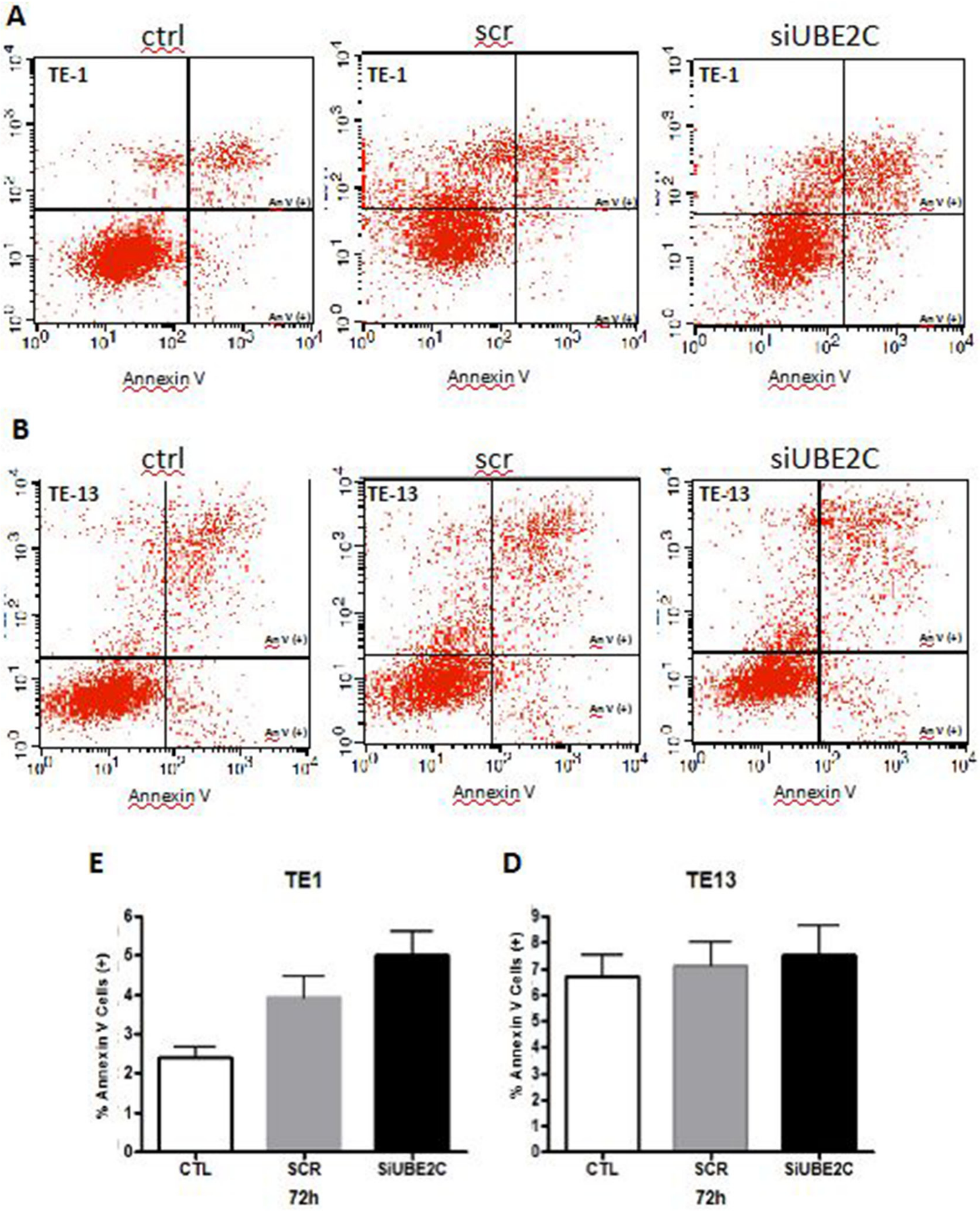

Supplementary Figure S3: ESCC cell lines Annexin V apoptosis assay. Representative dot-plot graph relative to Annexin-V fluorescence emission in TE-1 **A.** and TE-13 **B.** ESCC cells transfected with siRNA targeting UBE2C (siUBE2C) compared to those non-transfected (ctrl) and transfected with a siRNA scrambled sequence (scr). Graphical representation of the percentage of TE-1 **C.** and TE-13 cells **D.** positive to annexin-V.

Supplementary Table S1: Associations between esophageal squamous cell carcinoma (ESCC) patients overall survival and their clinicopathological characteristics and *UBE2C* gene expression

Parameter		Median	Time	Risk	95% CI	p value
UBE2C	Low	14.83	1.04	0.44	2.44	0.91
	High	12.77				
Age	<60	12.73	0.48	0.2	1.13	0.09
	>60	15.90				
Tumor Stage	Early	16.77	2.27	0.93	5.51	0.07
	Late	10.8				
Tumor Differentiation	Moderate	14.83	0.64	0.23	1.80	0.4
	Poor	11.1				
Tumor Site	Upper	3.53	2.58	1.02	6.50	0.01
	Medium / Lower	17.13				

Supplementary Table S2: Correlations between *UBE2C* gene expression and baseline characteristics of esophageal squamous cell carcinoma (ESCC) patients

Characteristics	≤ 0.002696	> 0.002696	*p* value
**Staging TNM**			
**I+II**	7 (43.7%)	9 (56.3%)	1.0000
**III+IV**	11 (45.8%)	13 (54.2%)	
**Histological Grade**			
**G1 +G2**	17 (43.6%)	22 (56.4%)	1.0000
**G3**	5 (38.5%)	8 (61.5%)	
**Tobacco Consumption**			
**Current Smoker**	18 (42.9%)	24 (57.1%)	0.7181
**Never Smoke**	4 (50.0%)	4 (50.0%)	
**Alcohol Consumption**			
**Current Drinker**	18 (42.9%)	24 (57.1%)	0.7181
**Never Drink**	4 (50.0%)	4 (50.0%)	
**Third**			
**Higher**	6 (50.0%)	6 (50.0%)	
**Medium**	7 (30.4%)	16 (69.6%)	0.1331
**Below**	5 (71.4%)	2 (28.6%)	

Supplementary Table S3: Univariate analysis of *UBE2C*mRNA expression impact on ESCC patients survival

Gene	Tumor Expression	Survival Time				P value
		Median (months)	Pattern Deviation	Confidence Interval 95%		
UBE2C	Low	12.934	1.764	9.477	16.391	0.79
	High	14.407	1.566	11.338	17.476	
